# Prognostic Impact of Caspase-8, CDK9 and Phospho-CDK9 (Thr 186) Expression in Patients with Uterine Cervical Cancer Treated with Definitive Chemoradiation and Brachytherapy

**DOI:** 10.3390/cancers14225500

**Published:** 2022-11-09

**Authors:** Maximilian Fleischmann, Ranadip Mandal, Izabela Kostova, Monika Raab, Mourad Sanhaji, Stephanie Hehlgans, Markus Diefenhardt, Claus Rödel, Emmanouil Fokas, Klaus Strebhardt, Franz Rödel

**Affiliations:** 1 Department of Radiotherapy and Oncology, Goethe-University, Theodor-Stern-Kai 7, 60590 Frankfurt am Main, Germany; 2 Frankfurt Cancer Institute (FCI), Goethe-University, Theodor-Stern-Kai 7, 60590 Frankfurt am Main, Germany; 3 Department of Gynecology, Goethe-University, Theodor-Stern-Kai 7, 60590 Frankfurt am Main, Germany; 4 German Cancer Research Center (DKFZ), Im Neuenheimer Feld 280, 69120 Heidelberg, Germany; 5 German Cancer Consortium (DKTK) Partner Site: Frankfurt, Im Neuenheimer Feld 280, 69120 Heidelberg, Germany

**Keywords:** caspase-8, CDK9, phospho-CDK9 (Thr 186), prognosis, cervical cancer, radiochemotherapy

## Abstract

**Simple Summary:**

Primary concurrent platinum-based chemoradiation (CRT) is the standard-of-care treatment for locally advanced cervical cancer. However, persistent, recurrent or metastatic disease remains a substantial cause of mortality in women worldwide. Biomarker research can help identify the potential mechanisms of chemo- and radioresistance, improve risk stratification and ultimately translate into novel treatment strategies. We report here that elevated pretreatment levels of caspase-8 and cyclin-dependent kinase 9 (CDK9) are associated with significantly improved relapse-free, distant metastasis-free and cancer-specific survival, while, in contrast, higher levels of phosphorylated CDK9 predict an increased risk of recurrence and distant metastases, and inferior cancer-specific survival.

**Abstract:**

**Introduction:** After primary platinum-based chemoradiation of locally advanced uterine cervical cancer, a substantial proportion of women present with persistent, recurrent or metastatic disease, indicating an unmet need for biomarker development. **Methods:** We evaluated the clinical records of 69 cervical cancer patients (Federation of Gynecology and Obstetrics, FIGO Stage > IB3) who were subjected to definitive CRT. Immunohistochemical scoring of caspase-8, cyclin dependent kinase 9 (CDK9) and phosphorylated (phospho-)CDK9 (threonine (Thr) 186) was performed on pretreatment samples and correlated with the histopathological and clinical endpoints, including relapse-free survival (RFS), distant metastasis-free survival (DMFS), cancer-specific survival (CSS) and overall survival (OS). **Results:** Lower levels of caspase-8 were more prevalent in patients with a higher T-stage (*p* = 0.002) and a higher FIGO stage (*p* = 0.003), and were significantly correlated with CDK9 expression (*p* = 0.018) and inversely with pCDK9 detection (*p* = 0.014). Increased caspase-8 levels corresponded to improved RFS (*p* = 0.005), DMFS (*p* = 0.038) and CSS (*p* = 0.017) in the univariate analyses. Low CDK9 expression was associated with worse RFS (*p* = 0.008), CSS (*p* = 0.015) and OS (*p* = 0.007), but not DMFS (*p* = 0.083), and remained a significant prognosticator for RFS (*p* = 0.003) and CSS (*p* = 0.009) in the multivariate analyses. Furthermore, low pCDK9 staining was significantly associated with superior RFS (*p* = 0.004) and DMFS (*p* = 0.001), and increased CSS (*p* = 0.022), and remained significant for these endpoints in the multivariate analyses. **Conclusion:** Increased caspase-8 and CDK9 levels correlate with improved disease-related outcomes in cervical cancer patients treated with CRT, whereas elevated pCDK9 levels predict worse survival in this patient population.

## 1. Introduction

Concurrent platinum-based chemoradiation (CRT) followed by high-dose rate brachytherapy (HDR-BT) is the standard-of-care treatment for women with locally advanced Federation of Gynecology and Obstetrics (FIGO) stage ≥ IB3 cervical cancer. This sequence provides high rates of local control of up to 90% [1,2] across all stages. For locally recurrent or persistent disease, potentially curative salvage hysterectomy or pelvic exenteration, if feasible, can improve survival and prevent a significant risk of morbidity [3]. In contrast, 15% to 61% of women with cervical cancer present with metastatic disease within the first 24 months after the completion of treatment, most of which is not curable [4,5]. Although doublet chemotherapy with a platinum derivate or topotecan and paclitaxel plus bevacizumab has significantly prolonged survival [6], intolerance, toxicity and disease progression have resulted in a large proportion of patients requiring novel treatment strategies beyond the first line. More recently, the programmed death 1 (PD-1) inhibitor pembrolizumab and the tissue factor-directed antibody and microtubule inhibitor drug conjugate tisotumab vedotin have been approved by the US Food and Drug Administration (FDA) as first-line and second-line treatment for recurrent or metastatic cervical cancer, respectively. The objective response rates (ORR) range between 15 and 24%, while the duration of the response often lasts only less than 10 months [7,8]. Therefore, biomarker research is addressing unmet needs in this patient population to identify high-risk settings; improve patient stratification; explore the potential molecular mechanisms of drug- and radioresistance, disease progression and metastasis; and ultimately establish new translational therapeutic approaches [9].

Caspase-8, a member of the caspase family of cysteine aspartate-specific proteases, is a central component of the extrinsic apoptotic pathway, as well as being involved in the intrinsic apoptosis pathway, anoikis, autophagy, necroptosis and pyroptosis [10,11,12]. Interestingly, we have previously demonstrated a novel pro-apoptotic function of caspase-8, where, upon CD95 stimulation, polo-like kinase 3 (Plk3) could phosphorylate caspase-8 at its Thr273 residue and stimulate its proapoptotic functions [13]. As a result, cancer cells regularly attempt to evade caspase-8-induced cell death through post-translational modifications [14,15,16], loss-of-function mutations of pro-apoptotic genes and the overexpression of anti-apoptotic genes or the expression of FLICE-like inhibitory protein long FLIPL [10,17,18]. Recent evidence has further highlighted the non-apoptotic properties of caspase-8, including its involvement in cell-cycle regulation, proliferation, angiogenesis, cell migration, cell invasion and resistance to chemotherapy [10,11,13,19]. As a result, the downregulation of caspase-8’s activity has been shown to be beneficial for several malignancies, which can be traced back to its non-apoptotic functions. 

We have recently also identified a novel mechanism where nuclear caspase-8 directly interacts with and inhibits the activity of cyclin-dependent kinase 9 (CDK9), thereby modulating RNA polymerase II (RNAPII)-mediated global transcription, including that of metastasis-associated genes [19]. Essentially, caspase-8 negatively regulates the autophosphorylation of CDK9 at Thr 186 (pCDK9), compromising its activation and the activity of the CDK9/CyclinT1-p-TEFb (Positive Transcription Elongation Factor b) complex. The latter phosphorylates the serine (Ser)2 residue of the YSPTSPS tandem repeats at the C-terminus domain (CTD) of RNAPII to promote transcriptional elongation [20]. As a result, loss of caspase-8 may lead to the hyperactivated state of CDK9, altering the transcriptional landscape of cervical cancer towards a more aggressive phenotype [19,21].

Here, we report a significant association between disease-related outcomes and pretreatment levels of caspase-8, CDK9 and pCDK9 (Thr 186) expression in a cohort of locally advanced cervical cancer patients treated with definitive CRT and BT.

## 2. Materials and Methods

### 2.1. Patient Characteristics

After institutional review board approval (approval number 31/15), 69 patients with histologically confirmed cervical cancer (FIGO Stages IB to IVA) treated with definitive CRT followed by HDR-BT between 1999 and 2017 at the Department of Radiotherapy and Oncology of the University Hospital (Goethe-University, Frankfurt am Main) were included in this retrospective study. All patients provided informed consent. Patients were routinely subjected to standard pretreatment staging, including computer or magnetic resonance tomography of the pelvis and abdomen, chest radiography and baseline laboratory parameters. 

### 2.2. Treatment and Follow-Up Assessment

Patients were treated with concomitant platinum-based CRT (40 mg/m^2^ weekly or 20 mg/m^2^/day 1–5, split course) with a median dose of 50.4 Gy (range: 45.0–66.6 Gy) delivered in daily fractions of 1.8 Gy using a photon beam linear accelerator (Elekta, Crowley, UK). Twelve patients additionally received two cycles of 5-Fluorouracil (600 mg/m^2^), and three patients received alternative chemotherapy regimens due to contraindications to platinum derivates. Pelvic external beam radiation therapy (EBRT) was applied using either a conventional four-field technique or intensity-modulated RT (IMRT). Initially, Point A-based BT (*n* = 41) was performed, which was later replaced by image-guided adaptive BT. The median overall equivalence dose in 2 Gy fractions (EQD2) generated by EBRT + BT for all patients was 106.2 Gy (range: 54.2–121.6 Gy). A minimum EQD2 of 78.1 Gy was applied to every patient who completed therapy. Twelve patients additionally received two cycles of 5-fluorouracil (600 mg/m^2^), two patients received mitomycin-C (7 mg/m^2^) or paclitaxel (25 mg/m^2^), and one patient received cisplatin in combination with gemcitabine (750 mg/m^2^). Follow-up examinations were scheduled every 3 months during the first 24 months after CRT, followed by 6 month intervals thereafter, and included gynecological examinations and imaging via pelvic magnetic resonance imaging (MRI) and/or abdominal computed tomography (CT).

### 2.3. Immunohistochemical Staining and Scoring

Formalin-fixed and paraffin-embedded (FFPE) pretreatment biopsy samples were subjected to a horseradish peroxidase technique (DAKO Envision Flex, Hamburg, Germany) with primary anti-caspase-8 (#PA5-87373, ThermoFisher, Dreieich, Germany), anti-CDK 9 (C12F7, Cell Signaling, Frankfurt am Main, Germany) and anti-phospho-CDK9 (Thr 186) antibodies (SAB4504223, Sigma Aldrich, Taufkirchen, Germany) applied at dilutions of 1:150, 1:150 and 1:50, respectively. Next, dextran polymer-conjugated horseradish peroxidase and 3,3′-diaminobenzidine (DAB) chromogen were used for visualization and a hematoxylin solution (Gill 3, Sigma Aldrich, Munich, Germany) was used for nuclei counterstaining. Negative control slides in the absence of primary antibodies were included for each staining procedure. To minimize interobserver variability, two investigators (F.R., I.K.), blinded to the patients’ clinical data, performed the evaluations. Next, the marker expression levels of caspase-8 and CDK 9 were dichotomized as being “high” (weighted score (WS) > 6) or “low” (≤6) based on the fraction of positive tumor cells (1, 0–25%; 2, 26–50%; 3, 51–75%; 4, >75%) and the staining intensity was characterized as 1+ (weak), 2+ (moderate) or 3+ (intense), as previously reported [22]. CDK9 phosphorylation (Thr 186) was scored as percentage of positive tumor cells, as noted via immunohistochemistry. Image acquisition for the analyses was performed using an AxioScanZ1 slide scanner and ZEN 2.1 software (Carl Zeiss, Jena, Germany).

### 2.4. Statistical Analysis

Correlation of the pathologic factors, including immunohistochemical scoring, was performed with Spearman’s correlation coefficients. The clinical endpoint pf local control was defined from the time of CRT initiation, with detection of the first local tumor or progression following CRT scored as a local failure event. Overall survival (OS) and cancer-specific survival (CSS) were defined as the time from the initiation of CRT to any level of disease progression (local, regional or distant), death for any reason or the day of the last follow-up, while cervical cancer-related death represented a CSS event. Survival curves were plotted according to the Kaplan–Meier method; log-rank testing and Cox proportional hazard modeling were applied for the univariate and multivariate survival analyses, respectively. Statistical tests were performed with an a priori α < 05 for significance using IBM SPSS Version 27 Software (IBM, Ehningen, Germany).

## 3. Results

### 3.1. Associations with Tumors and Patient Histopathologic Characteristics

**Figure 1:**
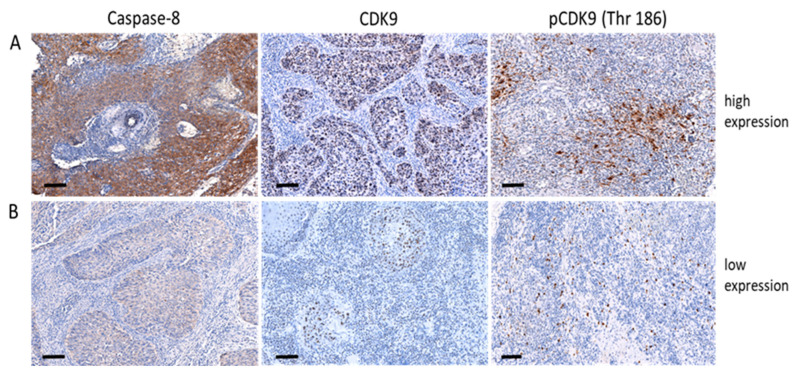
Examples of cervix cancer biopsies with immunohistochemical detection of high (**A**) and low (**B**) caspase-8, CDK9 and pCDK9 (Thr 186). Original magnification, 50×; scale bar, 100 μm. Casp 8, caspase 8; CDK9, cyclin-dependent kinase 9; pCDK9, phosphorylated (Thr 186) CDK9.

According to our assessment of pretreatment tumor biopsies, 42 patients (60.9%) presented with a low expression of caspase-8, 43 (62.3%) with low CDK9 and 39 (56.5%) with low pCDK9, while 27 (39.1%), 26 (37.7%) and 30 (43.5%) patients displayed elevated levels of caspase-8, CDK9 and pCDK9, respectively (Table 1). Figure 1 depicts examples of low and intensive (high) staining results for caspase-8, CDK9 and pCDK9. The weighted scores (WS) of the expression of caspase-8 and CDK9 revealed a significant correlation with pCDK9 signals in the corresponding tumor tissues (*p* = 0.05 and *p* = 0.01). 

Dichotomized scores (low vs. high) were based on the median weighted score (weighted score (WS): intensity of staining × % positive tumor cells) and the median of pCDK9-positive cells (%). Casp 8, caspase 8; CDK9, cyclin-dependent kinase 9; pCDK9, phosphorylated CDK9 (Thr 186). 

The median pCDK9 signal significantly increased in tumors with low caspase-8 expression (*p* = 0.018). Concerning patient- and tumor-related characteristics, lower levels of caspase-8 detection were more prevalent in patients with a higher T-stage (*p* = 0.002) and FIGO category (*p* = 0.003), whereas no significant differences were observed for age, N- and M-stage, tumor grading and p16^INK4a^ expression (Table 2). CDK9 detection was significantly increased (*p* = 0.014) in tumors with high caspase-8 expression but did not display a correlation with age, T-stage, N- and M-stage, tumor grading and FIGO category. Finally, we observed a significant negative correlation between high pCDK9 and low caspase-8 expression (*p* = 0.018), and a positive correlation (*p* = 0.019) with the level of CDK9 expression (Table 2).

### 3.2. Disease-Related Outcomes according to the Expression of Caspase-8, CDK9 and pCDK9

Next, we analyzed time-to-event data to evaluate the impact of detected caspase-8, CDK9 and pCDK9 on clinical endpoints. For the entire cohort, the median follow-up was 32 months (range: 5–174 months). The median OS was 76 months. The 2-year OS and 2-year CSS were 75.4% and 88.4%, respectively. Thirty-five (50.7%) patients died during follow-up; 18 (26.1%) due to cervical cancer, 14 (20.3%) from intercurrent disease and three (4.3%) because of treatment-related events. Following definitive CRT, 19 patients (25.6%) experienced disease relapse, including local recurrence (*n* = 8) and distant metastases (*n* = 10), of which 60% (*n* = 6) occurred in the first 24 months after the completion of treatment.

**Figure 2:**
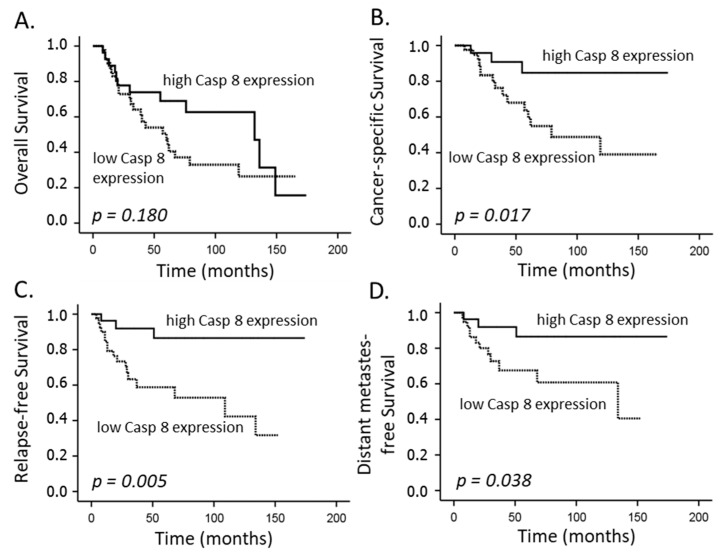
Overall survival (**A**), cancer-specific survival (**B**), relapse-free survival (**C**) and distant metastasis-free survival (**D**) according to caspase-8 expression (low: individual weighted score (WS) ≤ 6; high: individual WS > 6) in patients with cervical carcinoma treated with definitive CRT and BT.

**Figure 3:**
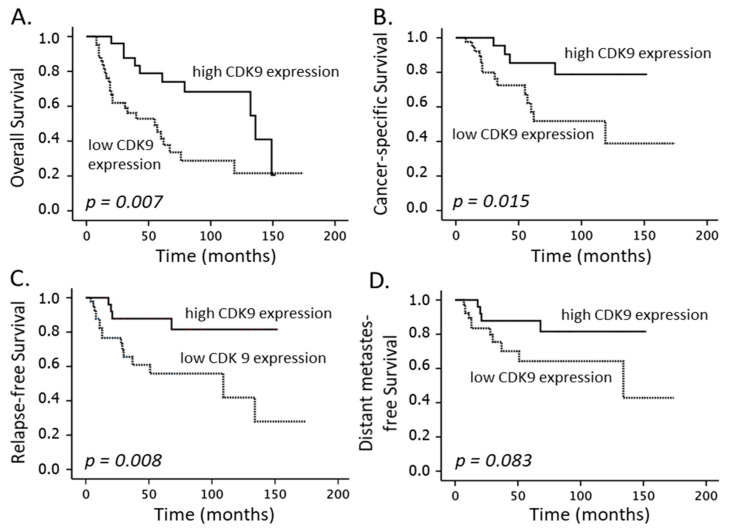
Overall survival (**A**), cancer-specific survival (**B**), relapse-free survival (**C**) and distant metastasis-free survival (**D**), according to cyclin-dependent kinase 9 (CDK9) expression (low: individual weighted score (WS) ≤ 6; high: individual WS > 6) in patients with cervical carcinoma cancer treated with definitive CRT and BT.

**Figure 4:**
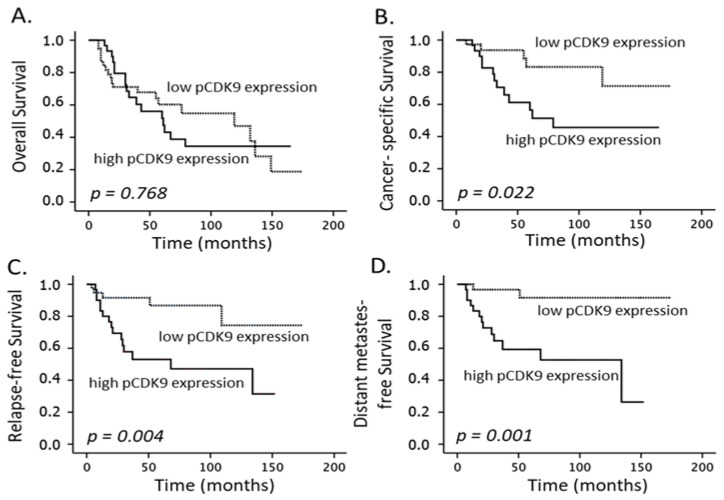
Overall survival (**A**), cancer-specific survival (**B**), relapse-free survival (**C**) and distant metastasis-free survival (**D**) according to phosphorylated (Thr 186) CDK9 (pCDK9) expression (low: ≤ median of 6; high: >median) in patients with cervical carcinoma treated with definitive CRT and BT.

Clinical factors with a significant impact on the CSS were the T-stage and FIGO category (*p* = 0.004 and *p* = 0.004, Table 3). Further, as depicted in Figure 2B, Figure 3B and Figure 4B, caspase-8 expression (*p* = 0.017), levels of CDK9 (*p* = 0.015) and, inversely, pCDK9 (*p* = 0.022) were associated with this endpoint in the univariate analysis, while the multivariate analyses revealed that CDK9 (*p* = 0.009) and pCDK9 (*p* = 0.009) remained significant independent predictors (Table 3). Finally, only CDK9 detection correlated significantly with the OS endpoint (*p* = 0.007).

## 4. Discussion

In the present analysis, we reported that elevated pretreatment detection of caspase-8 and CDK9 correlates with improved clinical endpoints (RFS, DMFS and CSS), whereas phosphoCDK9 (Thr 186) predicts an inverse prognostic profile in patients with cervical cancer treated with definitive CRT, suggesting the pro-metastatic properties of hyperactivated pCKD9.

Limited information is available on the expression patterns and functions of caspase-8 in cervical cancer, but there is evidence that caspase-8 activity gradually decreases from cervical intraepithelial neoplasia (CIN) to invasive cancer [23], which has been reported previously for other malignancies [24,25,26,27,28,29]. Loss of caspase-8, apart from its pro-apoptotic functions, may have an impact due to its non-apoptotic properties, facilitating migration and metastasis through multiple signaling pathways [29]. In previous studies by our group, low caspase-8 expression predicted unfavorable survival in anal and cervical cancer patients [22,30]. In addition, other groups reported that loss of caspase-8 activity was associated with cisplatin resistance in HEp-2 and non-small cell lung cancer (NSCLC) cell lines [31,32]. Nevertheless, the role of caspase-8 in tumor progression and response to therapy remains controversial [33]. Elevated nuclear expression of caspase-8 was recently reported to promote cleavage and inactivation of the ubiquitin-specific peptidase 28 (USP28), resulting in de facto p53 protein loss and cell cycle progression in a p53-proficient melanoma tumor model [34]. These findings are consistent with other reports in which elevated caspase-8 levels were correlated with therapy resistance and impaired outcomes in hepatocellular carcinoma and triple-negative breast cancer [28,35]. Here, we indicated that high caspase-8 expression was significantly correlated with improved local tumor control, lower numbers of distant metastases and long-term cancer-specific survival in a cohort of patients treated with definitive CRT plus BT, supporting its proposed tumor-suppressive activity [10]. Furthermore, the downregulation of caspase-8 expression or its catalytic activity have been shown to be beneficial for several cancers [27,28,29], but the molecular basis of the protease’s association with improved patient survival remains largely unexplored. It is, however, reasonable to speculate that this relationship may cover both cell death (apoptosis and necroptosis) and the non-apoptotic properties of the protease, including strong evidence for an essential role in suppressing the metastatic dissemination of cancer cells.

Given that low CDK9 expression is associated with increased apoptotic cell death, spontaneous DNA damage, genetic instability [36,37,38,39], and impacts on the efficacy of DNA DSB repair [37,40], one may assume that patients with low CDK9 expression have more aggressive disease. Indeed, here, we report that low histochemical CDK9 detection correlates with increased relapse as well as distant metastasis rates and unfavorable CSS, in both the uni- and multivariate analyses. As for caspase-8, the prognostic/predictive value of CDK9, however, still remains controversial among different tumor types. Whereas some authors have indicated that high levels of CDK9 expression in patients with pancreatic adenocarcinoma are associated with significantly shortened OS, especially in well differentiated tumors [36,41,42], others have demonstrated that higher CDK9 expression is associated with significantly improved survival [43,44]. 

Interactome analyses have indicated a novel interaction between caspase-8 and CDK9 in cervical cancer cell lines and patient-derived organoids [19], where the loss/attenuation of caspase-8 induces an overactivation of CDK9, which subsequently alters the cellular transcriptional landscape (transcriptional reprogramming) [19]. On a molecular level, caspase-8 allosterically inhibits the (auto)phosphorylation of CDK9 at Thr 186 (pCDK9) [45,46], thereby compromising its activation and, consequently, the activity of the CDK9/CyclinT1 complex-P-TEFb in phosphorylating the Ser2 residue at the C-terminal domain (CTD) of RNAPII [20]. This modulation in RNAPII’s activity results in altered cellular transcription, especially in the expression levels of several genes involved in migration and invasion, providing strong evidence for the substantial role of capsase-8 in suppressing metastasis in cervical cancer. Further, the knock-out/attenuation of caspase-8 resulted in a significant enhancement of 2D cell migration and 3D cell invasion in favor of the involvement of caspase-8 in the regulation of transcriptional metastases in cervical cancer [19]. As stated before, and in support of these mechanistic findings, for the first time, we report an inverse correlation between Thr 186 phosphorylation of CDK9 and caspase-8 and, as expected, with CDK9 immunoexpression, in the clinical setting. According to this, patients with an elevated level of pCDK9 revealed an impaired CSS and higher incidences of both local and distant failures, further confirming the pivotal role of the regulation of caspase-8 and CDK9 in the treatment response to CRT and BT.

We must acknowledge that the retrospective evaluation of the prognostic impact of caspase-8, CDK9 and pCDK9, and the relatively small cohort of patients and the small number of events represent the central limitations of our study and, therefore, selection bias cannot be excluded. In addition, technical advances (e.g., the implementation of image-guided adaptive BT) have further significantly impacted outcomes and survival, leading to a non-negligible potential for bias. Furthermore, the aging process of FFPE samples is known to affect the quality of immunohistochemical staining, which could have also affected the results. Accordingly, prospective validation of the impact of the expression of caspase-8, CDK9 and pCDK9 based on a standardized protocol for immunohistochemical evaluation of the proteins in samples from pretreated patients is required.

In summary, our data support the notion that increased pretreatment tumor levels of caspase-8 and CDK9 predict superior clinical responses in cervical cancer patients treated with definitive CRT plus BT, while higher levels of pCDK9 detection are predictive of an impaired clinical response. Beyond the association of these markers with prognosis and clinical treatment outcomes, these findings may further impact on future therapeutic decisions in cervical cancer. Ultimately, our recent translational investigations have revealed that caspase-8 knockout cancer cells and cervical cancer lines are more resistant to the small-molecule CDK9 inhibitor BAY1251152 in both 2D and 3D spheroid culture conditions. Further, combining the small-molecule inhibitor with cisplatin synergistically enhanced the sensitivity of caspase-8-deficient cancer cells to chemotherapeutic drugs [19]. These experiments demonstrated that the response of cervical cancer to cisplatin-based chemotherapy might be significantly improved by concurrently blocking CDK9. As several third-generation CDK9 inhibitors such as AZD-4573, BAY1251152 and JSH-150 are currently undergoing clinical trials, our findings emphasize CDK9 as a promising molecular target for treating cervical cancer patients, especially those with low caspase-8 expression [21]. However, stringent assessment and stratification of patients with high and low caspase-8 expression levels will be critical to evaluate the clinical success of future CDK9-based targeted strategies in cervical cancer and other tumor entities.

## Figures and Tables

**Table 1:** Results of immunohistochemistry for caspase-8, CDK9 and pCDK9 (Thr 186). WS = weighted score.

Marker	Casp 8 *n* (%)	CDK9 *n* (%)	pCDK9 *n* (%)
Dichotomized score	≤6 WS >6	≤6 WS >6	≤ median >
Low score	42 (60.9)	43 (62.3)	39 (56.5)
High score	27 (39.1)	26 (37.7)	30 (43.5)

**Table 2:** Clinicopathological findings according to the expression of caspase-8, cyclin-dependent kinase 9 (CDK9) and phosphorylated (Thr 186) pCDK9. Significant *p*-values are given in bold.

Marker	No.	Casp 8 Low *n* (%)	Casp 8 High *n* (%)	*p*-Value	No.	CDK9 Low *n* (%)	CDK9 High *n* (%)	*p*-Value	No.	pCDK9 Low *n* (%)	pCDK9 High *n* (%)	*p*-Value
**Age**												
≤ 59 years	38	21 (30.4) 21(30.4)	17 (24.6)		38	24 (34.7)	14 (20.2)		38	22 (31.8)	16 (23.3)	
> 59 years	31		10 (14.6)	0.291	31	19 (27.6)	12 (17.4)	1	31	17 (24.6)	14 (20.2)	0.799
**T-Stage**												
T1/2	35	15 (27.7)	20 (30.0)		35	18 (26.1)	17 (24.6)		35	21 (30.4)	14 (20.3)	
T3/4	34	27 (39.1)	7 (10.2)	**0.002**	34	25 (36.3)	9 (13.0)	0.058	34	18 (26.0)	16 (23.3)	0.554
**N-Stage**												
N0	35	23 (33.3)	13 (18.8)		35	20 (28.9)	15 (27.7)		35	21 (30.4)	14 (20.2)	
N1	33	19 (27.6)	14 (20.3)		33	20 (28.9)	10 (15.8)		33	18 (26.0)	15 (21.8)	
Nx	1	1 (1.5)		0.653	1		1 (1.5)	0.244	1		1 (1.5)	0.467
**M-Stage**												
M0	59	36 (52.2)	24 (34.7)		59	35 (50.7)	24 (34.7)		59	36 (52.2)	23 (33.3)	
M1	10	7 (10.2)	3 (4.3)	0.522	10	8 (11.7)	2 (2.9)	0.212	10	3 (4.3)	7 (10.2)	0.067
**FIGO**												
Low (IA–IIB)	26	10 (15.8)	15 (27.7)		26	14 (20.6)	14 (20.6)		26	18 (26.0)	8 (11.6)	
High (IIIa–IVA)	43	32 (46.4)	11 (15.9)	**0.003**	42	29 (42.6)	13 (19.2)	0.136	43	21 (30.4)	22 (31.8)	0.098
**Grading**												
G1/2	36	21 (30.4)	15 (27.7)		36	21 (30.4)	15 (27.7)		36	18 (26.0)	18 (26.0)	
G3	31	20 (29.9)	11 (15.9)		31	22 (31.8)	9 (13.0)		31	19 (27.6)	12 (17.5)	
Gx	2	1 (1.5)	1 (1.5)	0.831	2		2 (2.9)	0.095	2	2 (2.9)		0.294
**p16^Ink4a^**												
Low (WS ≤ 6)	26	18 (26.0)	8 (11.6)	0.268	25	19 (27.9)	6 (8.8)	0.065	26	16 (23.1)	10 (14.5)	0.513
High (WS > 6)	43	24 (34.8)	19 (27.5)		43	23 (33.8)	20 (29.4)		43	23 (33.3)	20 (28.9)	
**Casp 8**												
Low (WS ≤ 6)					42	31 (44.9)	11 (15.9)		42	19 (27.7)	23 (33.3)	
High (WS > 6)					27	12 (17.4)	15 (27.7)	**0.014**	27	20 (30.0)	7 (17.9)	**0.018**
**CDK9**												
Low (WS ≤ 6)	43	31 (44.9)	12 (17.5)						43	29 (42.0)	14 (20.3)	
High (WS > 6)	26	11 (15.9)	15 (27.7)	**0.014**					26	10 (14.5)	16 (23.2)	**0.019**
**pCDK9**												
Low (≤med)	39	19 (27.7)	20 (30.0)		39	29 (42.0)	10 (14.5)					
High (>med)	30	27 (39.1)	7 (10.3)	**0.018**	30	14 (20.3)	16 (23.2)	**0.019**				

**Table 3:** Univariate and multivariate analyses of prognostic factors in patients with cervical carcinoma treated with CRT and BT.

			Multivariate Analyses		
		95% Confidence Interval			
	Univariate *p*-Value	Hazard Ratio (HR)	Lower	Upper	*p*-Value
**Relapse-free survival**					
T-stage (T1–2/T3–4)	**0.004**	3.03	1.07	8.58	**0.036**
FIGO (IA–IIB/IIIA–IVA)	**<0.001**	2.18	0.33	14.42	0.415
Casp 8 (WS ≤ 6/> 6)	**0.005**	1.32	0.31	5.47	0.702
CDK 9 (WS ≤ 6/>6)	**0.008**	5.48	1.95	15.97	**0.003**
pCDK 9 (≤/> median)	**0.004**	4.56	1.48	13.97	**0.001**
**Distant metastasis-free survival**					
T-stage (T1–2/T3–4)	**0.005**	4.15	1.16	14.8	**0.028**
FIGO (IA–IIB/IIIA–IVA)	**0.001**	3.47	0.3	39.92	0.318
Casp 8 (WS ≤ 6/> 6)	**0.038**	1.25	0.28	5.54	0.768
pCDK 9 (≤/> median)	**0.001**	7.33	1.64	32.76	**0.009**
**Cancer-specific survival**					
T-stage (T1–2/T3–4)	**0.004**	2.83	0.9	8.84	0.073
FIGO (IA–IIB/IIIA–IVA)	**0.004**	1.53	0.19	11.8	0.698
Casp 8 (WS ≤ 6/>6)	**0.017**	1.1	0.25	4.85	0.898
CDK 9 (WS ≤ 6/>6)	**0.015**	4.75	1.17	15.32	**0.009**
pCDK 9 (≤/> median)	**0.022**	4.18	1.43	12.16	**0.009**

Abbreviations: Casp 8, caspase 8; CDK9, cyclin-dependent kinase 9; pCDK9, phosphorylated (Thr 186) CDK9; FIGO, Federation of Gynecology and Obstetrics. Significant *p*-values are given in bold.

## Data Availability

Not applicable.
